# Numerical study of granular flow in a slit funnel with a novel structure to avoid particle clogging

**DOI:** 10.1371/journal.pone.0286591

**Published:** 2023-06-02

**Authors:** Yi Peng, Sheng Zhang, Jiangfeng Wan, Yangyang Yang, Kewei Tao, LiDong Ma, Guanghui Yang, Lei Yang, Mengke Wang

**Affiliations:** 1 Institute of Modern Physics, Chinese Academy of Sciences, Lanzhou, China; 2 University of Chinese Academy of Sciences, Beijing, China; 3 Center for Basic Teaching and Experiment, Nanjing University of Science and Technology, Jiangyin, China; 4 Interdisciplinary Center for Fundamental and Frontier Sciences, Nanjing University of Science and Technology, Jiangyin, China; 5 School of Nuclear Science and Engineering, East China University of Technology, Nanchang, China; Sivas Cumhuriyet University, TURKEY

## Abstract

To solve the problem of particle clogging in slit funnels and to obtain a stable discharge flow rate, we proposed a new funnel structure, namely the slit baffle funnel. We conducted a systematic investigation using the discrete element method (DEM) to study the effects of funnel half-angle *θ*, outlet width *W*, and baffle height *H* on flow rate and flow pattern. We found that the proposed structure could effectively avoid particle clogging and guarantee a continuous and stable flow rate with small outlet width. Under the condition of *H >3 d*, a bigger flow rate was obtained at a smaller funnel half-angle. This new funnel structure could be applied to solve clogging problems associated with granular matter in the slit geometry in mining, agriculture, food, and pharmaceuticals.

## 1. Introduction

Granular media is a complex system composed of a large number of discrete particles, which are widely distributed throughout nature, such as sand dunes [1] and avalanches [2–4], and also found in mining and powder industries [5]. Characterized by strong dissipation, microscopic anisotropy, and complex phase transition behavior, in recent decades, it has been challenging to describe granular media with several new tools in progression [6–8]. The study of granular media could help us to better understand the properties of nonideal fluids and dissipative systems [9].

Silo flow refers to the gravity-driven flow of granular media that are discharged from a silo through an orifice. This flow has been studied extensively because of its wide application in industrial areas, such as mining, agriculture, food, and pharmaceuticals [10–13]. In silo flow, the flow behavior of granular materials is spatially nonuniform, and according to the flowing behaviors, the whole space can be divided into funnel flow region, free flow region, and quasi-static filling region [14].

Due to the complex rheological behavior of silo flow, it is difficult to form a systematic theory to describe the flow regimes. Recently, Barker et al. obtained the novel exact solution of vertical flow using a linear version of the ‘*μ*(*I*),*Φ*(*I*)-rheology’ to achieve a great prediction and evaluation of the flow rate of the hopper-fed process [15]. To date, the general empirical formula of flow rate for silo flow has been proposed according to an investigation of the flow rate at the circular outlet of a three-dimensional flat-bottomed silo [16]. Subsequently, numerous experiments showed that this formula could be applied universally for different materials, geometries of particles, and funnel structures, which is as follows:

$$Q=C\rho_{b}\sqrt{g}{\left(D_{0}-kd\right)}^{5/2},$$ (1)

Where *Q* is the particle mass flow rate, *ρ*_*b*_ is the bulk density of the particle, *g* is the gravitational acceleration, *D*_0_ is the diameter of the orifice, *d* is the particle diameter, and *C* and *k* are empirical constants related to the material properties, such as friction coefficient, bulk density and the geometrical shape of the particle and the outlet, which commonly are determined experimentally [17, 18]. Numerous studies have found that funnel half-angle and the shape of orifice have a significant influence on funnel flow rate [19–23].

For different funnel half-angles, Rose and Tanaka [24] proposed a multiplicative factor *F*, which could be incorporated into Eq (1) as follows:

$$F=\left\{\begin{array}{l} 1,\;\tan\;\alpha \tan\;\chi \geq 1\\ {\left(\tan\;\alpha \tan\;\chi \right)}^{-0.35},\tan\;\alpha \tan\;\chi <1 \end{array}\right.,$$ (2)

where *α* is the funnel half-angle, *χ* is notionally the angle between the flowing zone boundary and the horizontal boundary, and a value of 45° is recommended by the Draft British Code of Practice for Silo Design in the absence of more reliable information [25]. For the rectangular outlet of a three-dimensional flat bottom silo [26], the equation is as follows:

$$Q=C\rho_{b}\sqrt{g}\left(L_{0}-kd\right){\left(W_{0}-kd\right)}^{3/2},$$ (3)

where *L*_0_ is the length of the outlet, *W*_0_ is the width of the outlet, and *C* takes 1.03 when *W*_0_ ⪡ *L*_0_ [17].

To study the flow regimes and phase transition, a simplified quasi-two-dimensional silo has been widely adopted. Because the width between the two plates is usually less than 1.5 times of the particle diameter, the loading process will be disrupted because of the potential clogging, leading to a limit of the repeatability of physical experiments. In research studies and industry applications, there are many similar structures. Recently, research group of the Institute of Modern Physics proposed a target for neutrino generation composed of particle jet discharge from a silo with a slit outlet and analyzed the dynamics and the flow rate with varied outlet widths of particle jet [27, 28]. To study the conditions for a continuous flow rate during the loading process of various agricultural seeds and plastic pellets, Davies and Desai [29] studied the relationship between the width of the horizontal orifice and the diameter of the wheat and barley. When the width of horizontal orifice is between 1.9 and 2.7 *d*, the orifice will be blocked.

With a small outlet size, particles usually form an arch at the funnel outlet, which leads to clogging [30–32]. For a three-dimensional funnel, a critical outlet size exists, and when the outlet exceeds that size, the clogging probability decreases significantly [33]. This clogging could be broken by inducing vibrations or jet airflow, followed by restarting the flow. For two-dimensional funnels, because of the long experiment period, the critical outlet size remains controversial. Recently, a silo with multiple outlets demonstrated a low clogging probability, likely because the arch above one outlet was disrupted by the flow through another outlet [34–36].

In this work, we designed a new funnel structure, namely slit baffle funnel, to solve the clogging problem in the slit funnel and to obtain a continuous flow rate. For clarity, we conducted a systematic investigation of the effects of funnel half-angle, outlet width, and baffle height on flow rate, and found that a stable and continuous flow rate existed with a small outlet width. We also assessed the availability of the slit baffle funnel in engineering applications.

## 2. Material and method

### 2.1 Discrete element method

**Fig 1:**
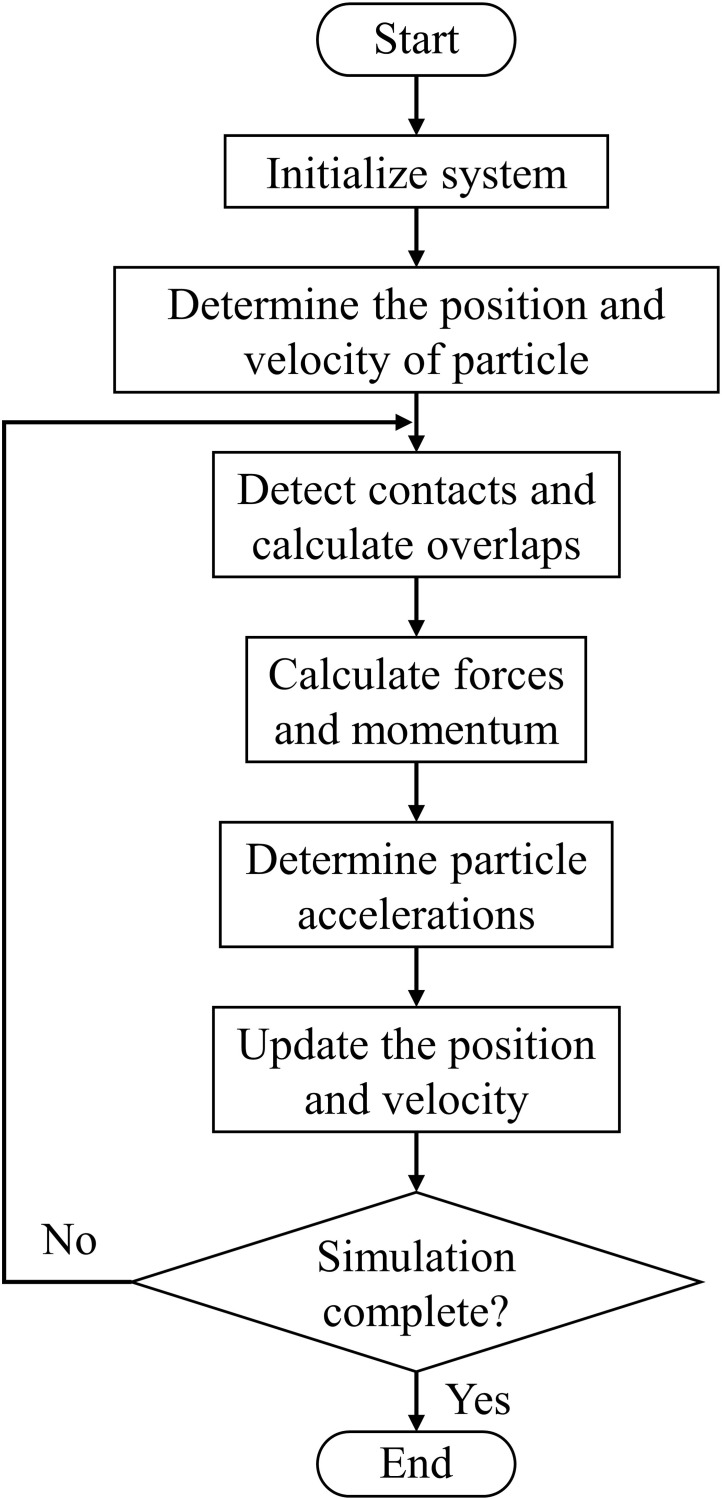
Flowchart of the DEM algorithm.

The discrete element method (DEM) is a complementary method to the finite element method used to solve complex problems in engineering and applied sciences and to study the natural flow phenomena of particles of different shapes [37, 38]. DEM uses the Newtonian equation to track each contact of particles to describe the contact force, rotation, momentum, and displacement of each particle to obtain parameters that are difficult to obtain under experimental conditions. These parameters, including particle trajectory, transient force, and the dynamic process of force balance between particles, can be used to describe the interaction of particles [39–42]. Zhu et al. described the solving process of DEM in detail and divided it into five phases: (1) initialing integration to determine the position and velocity of particles, (2) detecting the contacts between the elements and calculating the overlaps, (3) calculating the interaction forces and momentum, (4) determining the accelerations, and (5) integrating again to update the motion. The process of these five phases is repeated until the simulation is completed [43]. And the solving process was presented using flowchart in Fig 1. In this study, we used a multi-GPU-DEM program developed by our group, and the specific method referred to previous articles published by the group [44, 45].

### 2.2 Material

**Fig 2:**
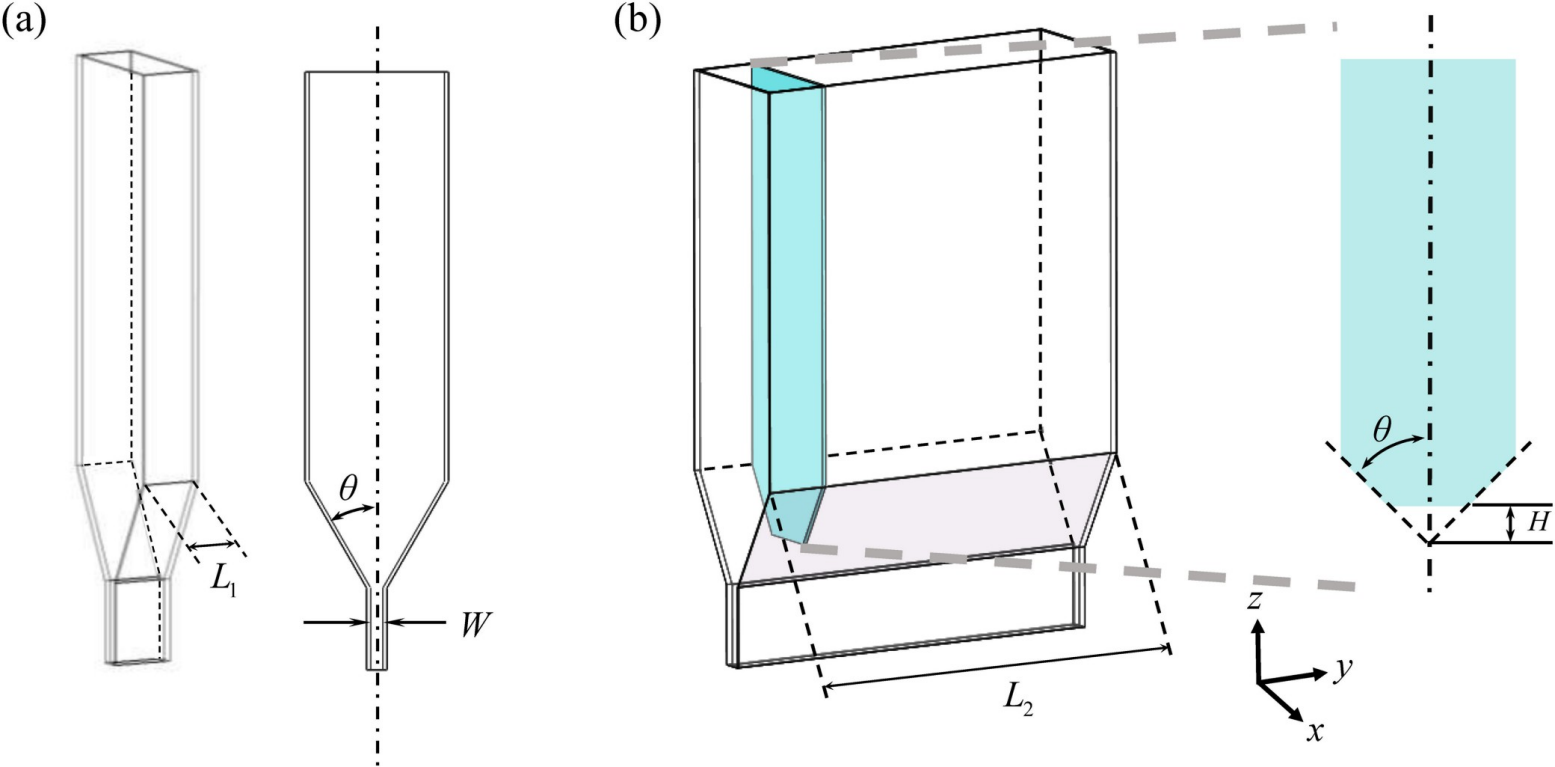
Schematic diagram of the funnel. (a) slit funnel;(b) slit baffle funnel.

Two funnel structures were used in the simulation. Structure I (the slit funnel) as shown in Fig 2(A), had an outlet length *L*_1_ of 150.00 *mm* (25 *d*), which ensured that the particles would not cause clogging in the slit direction. Structure II (the slit baffle funnel) as shown in Fig 2(B), was based on the slit funnel by extending the length to *L*_2_ and adding a baffle vertically at *L*_1_ with a baffle height of *H*. The length of *L*_2_ was long enough to ensure that the particles did not reach the border of the funnel after washing out from the bottom of the baffle and did not cause clogging. The geometric properties of the funnels are presented in Table 1.

**Table 1:** Funnel geometry parameters.

Quantity	Symbol	Value
Funnel outlet width [*mm*]	*W*	6.6, 7.2, 7.8, 8.4, 9.0, 9.6, 10.2, 10.8, 11.4, 12.0, 12.6, 15, 18, 24, 30, 60
Length of slit funnel [*mm*]	*L*_1_	150
Length of slit baffle funnel [*mm*]	*L*_2_	550
Funnel half-angle [°]	θ	30, 45, 60
Baffle height [*mm*]	*H*	6,12,15,16,18,24,30,36,42,48,54,60

Mono-size plexiglass particles with bulk density *ρ* = 1.19 *g/cm*^*3*^ and diameter *d* = 6.00 *mm* were loaded in this work. The parameters of particle material properties are presented in Table 2. To obtain a stable flow rate, we loaded 400,000 particles with a funnel outlet width *W* = 10 *d*. With *W* = 4 *d* and 5 *d*, we loaded 300,000 particles and otherwise, we loaded 200,000 particles. For the calculation, we used a step size of 1e^-5^, and packed the particles for 3 *s*, and then opened the funnel outlet and continued the operation for 10 *s*.

**Table 2:** Particle parameters.

Quantity	Symbol	Value
Particle diameter [*mm*]	*D*	6.00
Particle density [*g/cm*^3^]	*ρ*	1.19
Poisson ratio	υ	0.22
Restitution coefficient	e	0.93
Friction coefficient	*μ*	0.40

To investigate the clogging of the funnel and to ensure the accuracy of flow data statistics, several simulations were conducted for the case of the small funnel outlet width *W*. The number of simulation times was *N* > 10 for each event, and the flow rate of the event was obtained by averaging the data for each simulation and calculating the statistical error *E*. For each funnel outlet width *W*, we counted the number of clogging events (*N*_*a*_) of the funnel to obtain the clogging probability *J*(*d*), where *J*(*d*) = *N*_*a*_*/N*, and defined the maximum outlet width of funnel clogging as the critical outlet width *W’*.

The particle mass flow rate used in this study refers to the mass of particles discharging from the funnel outlet per unit time in grams per second (*g/s*). All subsequent physical lengths in this study are related to particle diameter; therefore, unless specifically mentioned, all physical lengths are expressed in units of the particle diameter *d*.

## 3. Results and discussions

### 3.1 Flow rate in slit funnel

**Fig 3:**
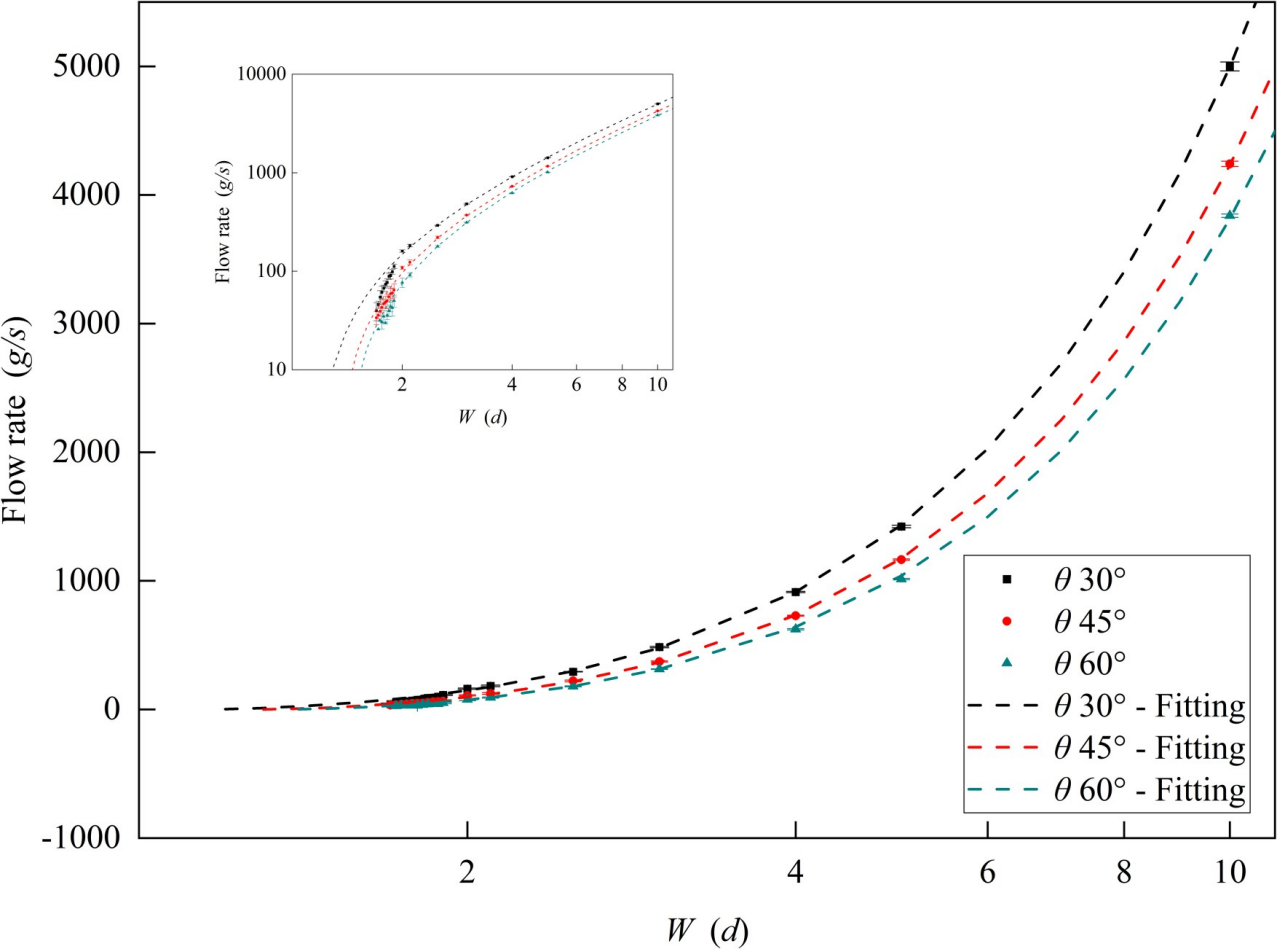
The relationship between flow rate and funnel outlet width and funnel half-angle. The funnel outlet width *W* ranges from 1.1 to 10 *d* and the funnel half-angle *θ* is equal to 30°, 45°, and 60°.

In this section, the effects of funnel outlet width *W* and funnel half-angle *θ* on the flow rate was investigated for a slit funnel with outlet length *L*_1_ of 25 *d*. As shown in Fig 3, the particle flow rate conformed to Eq (3), which was modified by the multiplicative factor *F* for different funnel outlet widths and funnel half-angles. The flow rate increased as the funnel outlet width *W* increased and, conversely, it decreased as the funnel half-angle *θ* increased. As shown in Fig 3, when the funnel outlet width *W* < 1.9 *d*, the flow rate was significantly lower than the equation fitting result and was unstable. When outlet width *W* < 1.7 *d*, the funnel was completely clogged and the flow rate was cut off.

**Fig 4:**
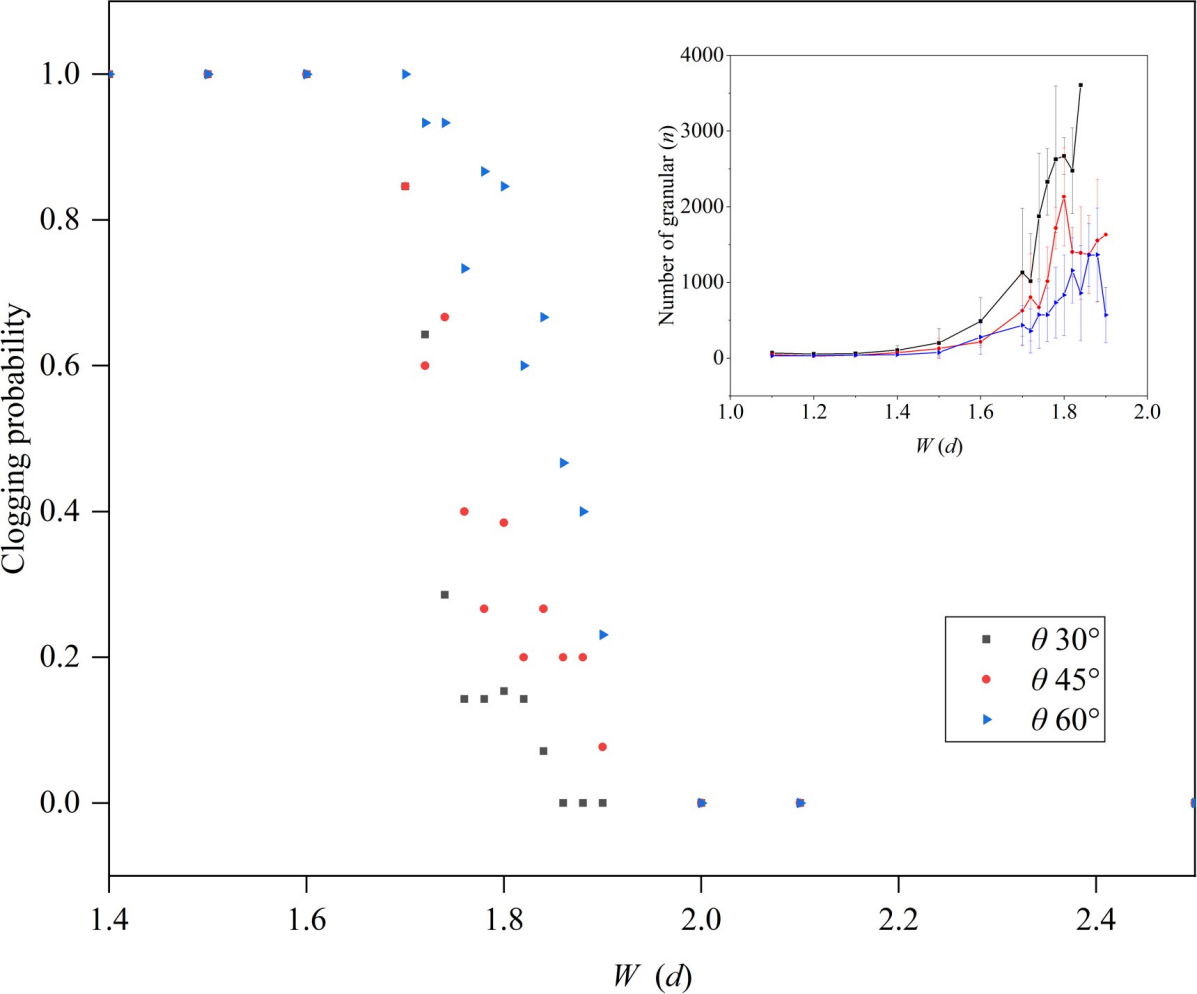
The clogging probability *J*(*d*) in a slit funnel. Funnel half-angle *θ* is equal to 30°, 45°, and 60°. The illustration shows the total number of particles discharged from the funnel before the funnel clogged.

As shown in Fig 4, the clogging probability *J*(*d*) of the funnel was counted for different outlet widths. When the funnel outlet width *W* < 1.7 *d*, *J*(*d*) = 1 and the funnel was completely clogged. When 1.7 ≤ *W* ≤ 1.9 *d*, *J*(*d*) decreased sharply from 1 to zero. Finally, when *W* > 1.9 *d*, *J*(*d*) was equal to zero and the funnel was not clogged. For the slit funnel with a half-angle *θ* of 30°, the critical outlet width *W’* was equal to 1.85 *d*. We counted the number of particles discharged from funnel outlet before clogging and found that the number of particles gradually increased with funnel outlet width, and strong fluctuations occurred when 1.7 ≤ *W* ≤1.9 *d*.

### 3.2 Flow rate in slit baffle funnel

Various methods and geometric structures have been attempted to achieve continuous stability of the loading process in quasi-two-dimensional funnel experiments. We found that a continuous and stable flow rate existed with the addition of a baffle in the slit funnel, for which a systematic study was conducted.

**Fig 5:**
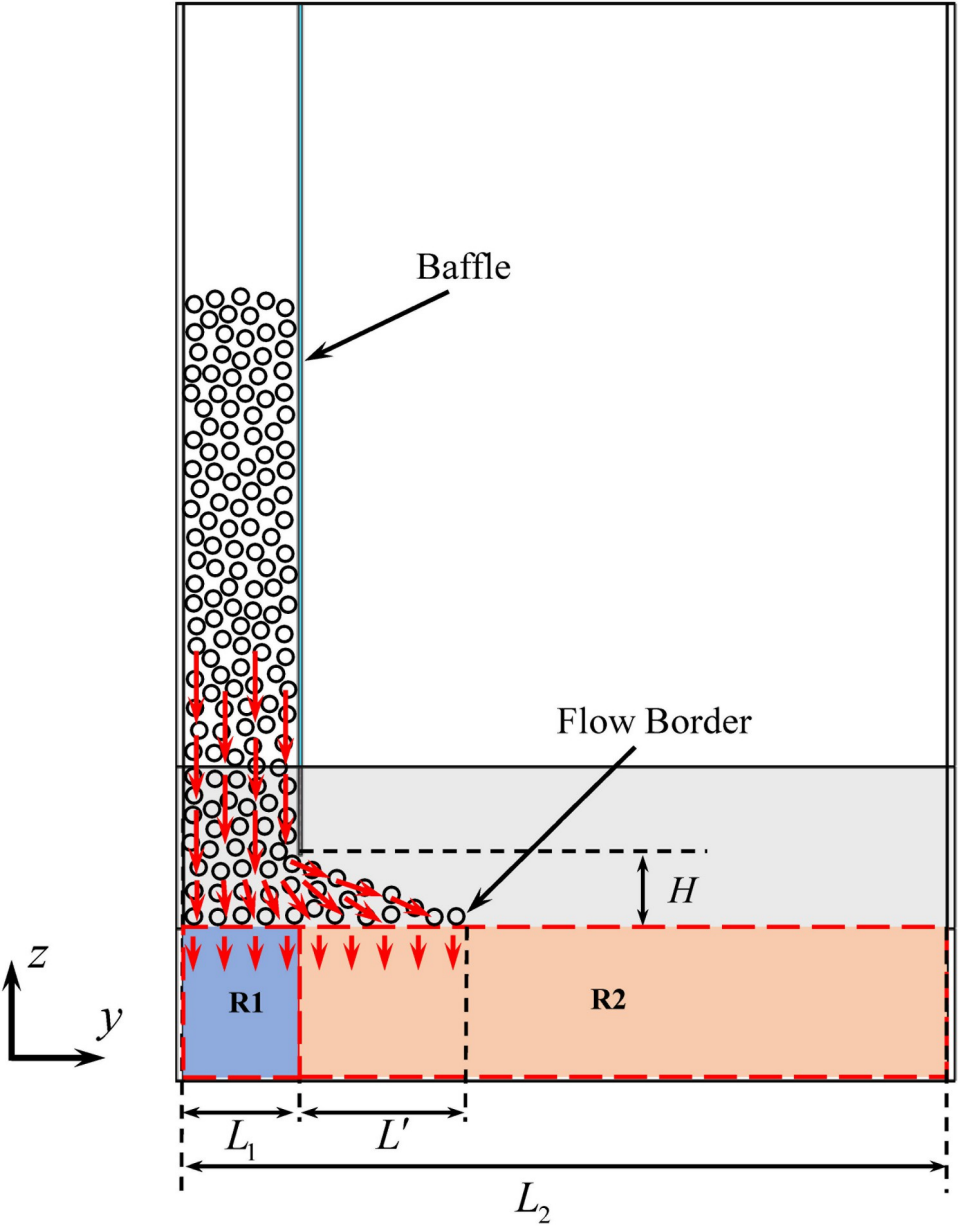
Side view of slit baffle funnel. The red arrow is the direction of particle flow.

We extended the length of structure I (slit funnel) to *L*_2_, and added a baffle vertically at *L*_1_ to construct funnel structure II: the slit baffle funnel. We divided this funnel into two regions, Region 1 (R1) and Region 2 (R2), using the baffle as the boundary, where the length of R1 was same as that of the slit funnel. As shown in Fig 5, particles were loaded in R1 in the simulation and the value of baffle height *H* was taken to 10 *d* to ensure that particles could flow from the bottom of the baffle to R2. The flow rates in the two regions were counted separately, and we investigated the influence of the outlet width *W* and the half-angle *θ* of the slit baffle funnel on the flow rate.

**Fig 6:**
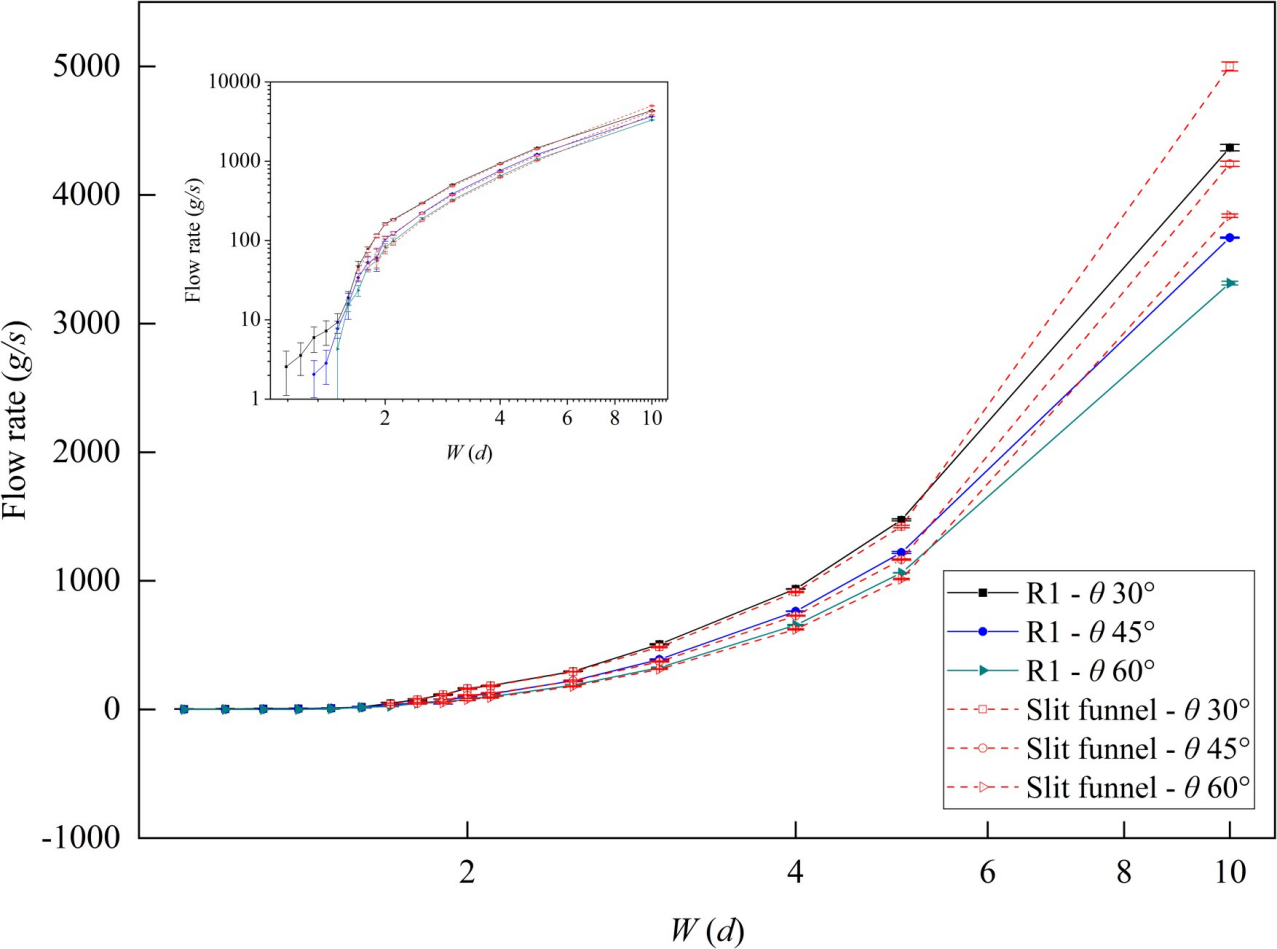
The flow rate of R1 in the slit baffle funnel. Where *H* = 10 *d* and the red dotted line is the flow rate of slit funnel.

As shown in Fig 6, the flow rate of R1 in the slit baffle funnel was consistent with the flow rate in the slit funnel, but the flow rate of R1 was slightly lower than the slit funnel when outlet width *W* = 10 *d*. Notably, the addition of the baffle to the slit funnel effectively reduced the critical outlet width for funnel clogging. This critical outlet width was influenced significantly by the funnel half-angle. When *θ* = 60° and *W* < 1.6 *d*, the funnel would clog. Similarly, when *θ* = 45° and *W* < 1.3 *d*, the funnel also would clog. When *θ* = 30°, however, the funnel still had a flow rate even if *W* = 1.1 *d*, and the critical outlet width *W’*<1.1*d*. In the case of small outlet widths, when *θ* < 30°, an intermittent flow rate of R1 existed.

**Fig 7:**
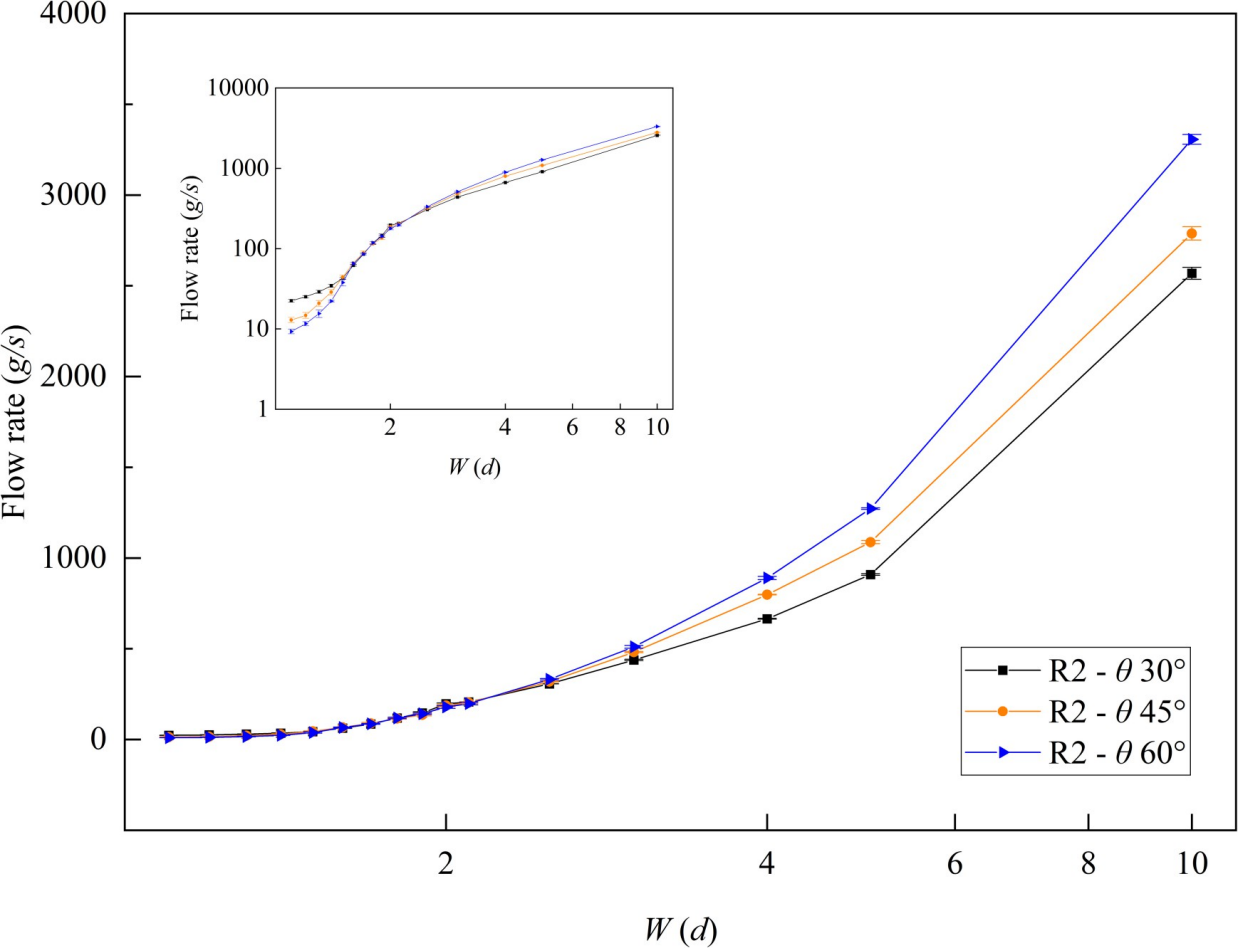
The flow rate of R2 in the slit baffle funnel. Where *H* = 10 *d*.

As shown in Fig 7, R2 had a stable and continuous flow rate with an error *E* below 5.0% for the small outlet width. When outlet width *W* < 2.5 *d*, the R2 flow rate decreased with an increase in the funnel half-angle *θ*, and in the case of the funnel half-angle *θ* = 30°, the funnel had a great flow rate, and the stable flow rate could reach 24.16 *g/s* at *W* = 1.1 *d*. By contrast, when the outlet width *W* > 2.5 *d*, the flow rate increased with an increase in the funnel half-angle *θ*.

**Fig 8:**
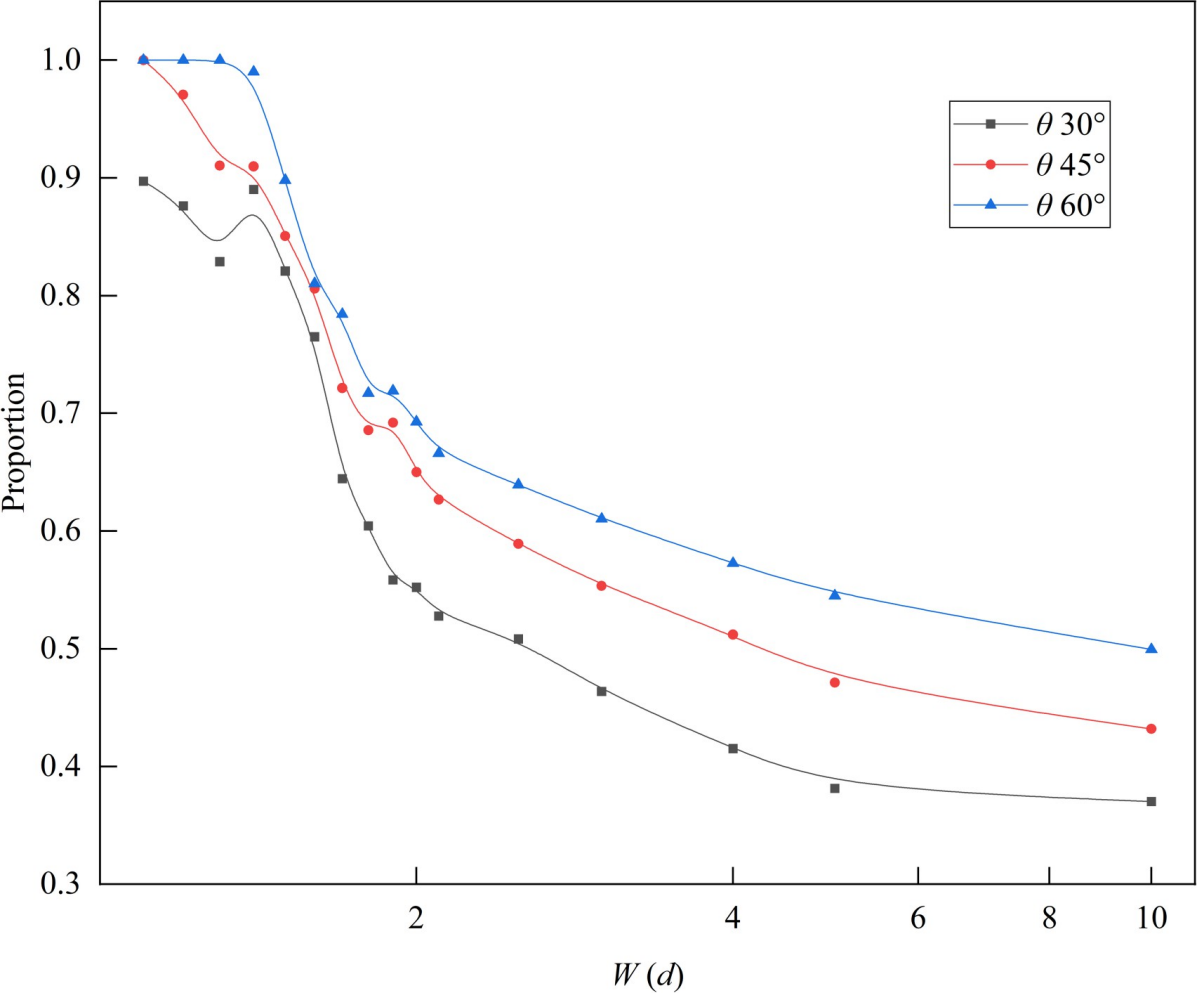
The proportion of R2 flow rate in the slit baffle funnel out of the total flow rate. The value of baffle height *H* is taken as 10 *d*.

We analyzed the proportion of R2 flow rate in the total flow rate, as shown in Fig 8. When the funnel outlet width *W* < 1.8 *d*, the proportion was higher than 0.6. As the funnel outlet width decreased, the proportion gradually increased and exceeded 0.8 when the outlet width *W* was less than 1.6 *d*. The proportion was significantly influenced by the funnel half-angle *θ*, and increased with increasing *θ*.

**Fig 9:**
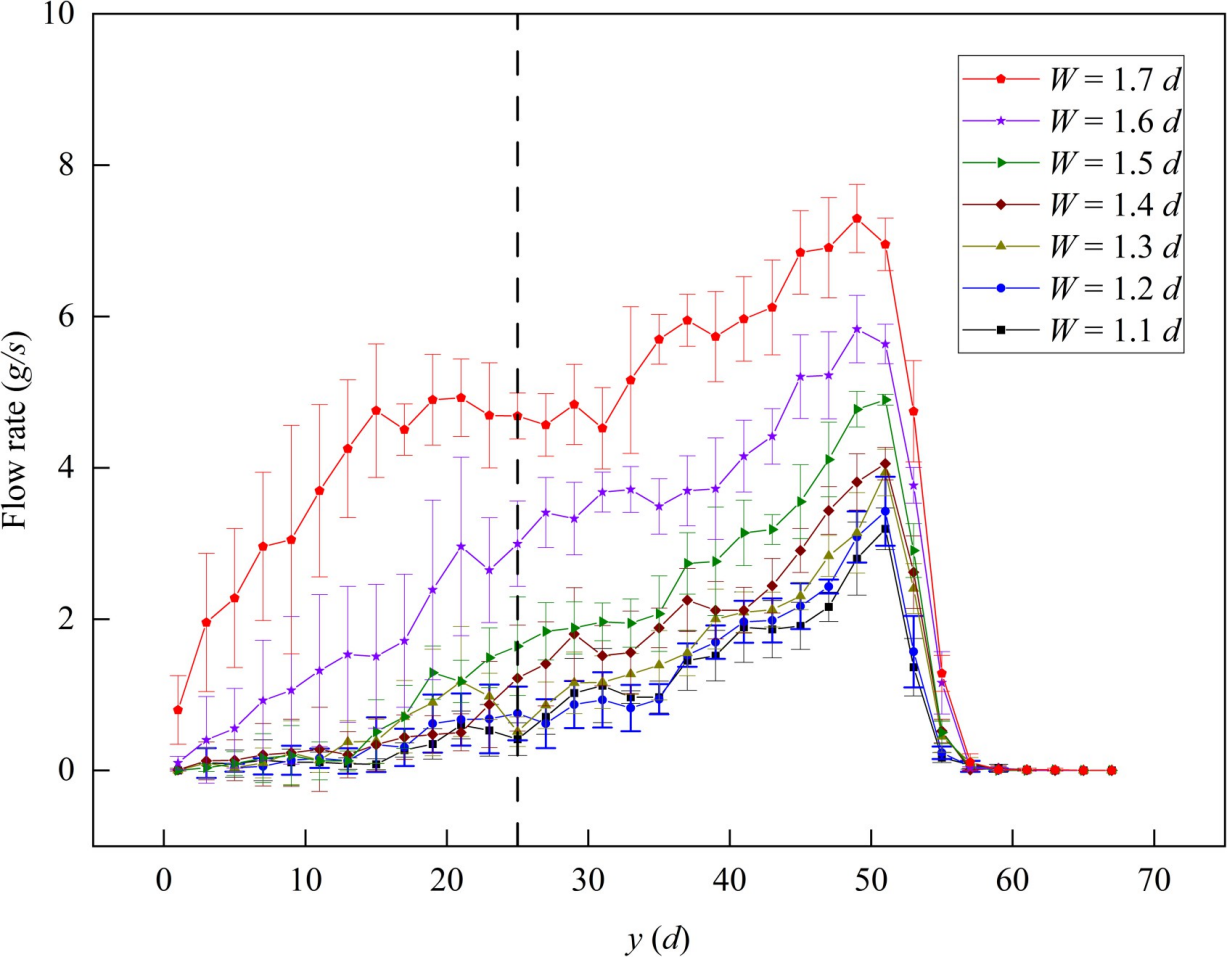
Detail flow rate in the slit baffle funnel along the *y*-direction. Where the funnel half-angle *θ* is 30° and the baffle height *H* is 10 *d*. The black dashed line indicates the baffle.

When the slit baffle funnel outlet width *W* was small, the R1 flow rate fluctuated considerably. Thus, we divided the funnel into multiple regions along the funnel slit direction, that is, the *y*-direction, with the length unit *l* = 2*d*, as shown in Fig 5. The position where the particle flow ended in the *y*-direction in the funnel was defined as the particle flow boundary, and the length between the baffle and the particle flow boundary was defined as the R2 flow zone length *L’*. The flow rate of each region was counted separately, as shown in Fig 9, and we found that the flow rate increased gradually with the *y*-direction and reached the maximum near the particle flow boundary.

To the left of the baffle (i.e., R1), the flow rate fluctuated greatly and was close to zero when the outlet width *W* < 1.6 *d* and in the region of *y* < 15 *d*. In comparison, R2 flow rate was significantly larger than R1 flow rate and was more stable.

Figs 8 and 9 show that for small outlet widths (i.e., *W* ≤ 1.7 *d*), the flow rate in the slit baffle funnel was concentrated in R2, and at the particle flow boundary, the flow rate reached its maximum.

### 3.3 Velocity field in slit baffle funnel

**Fig 10:**
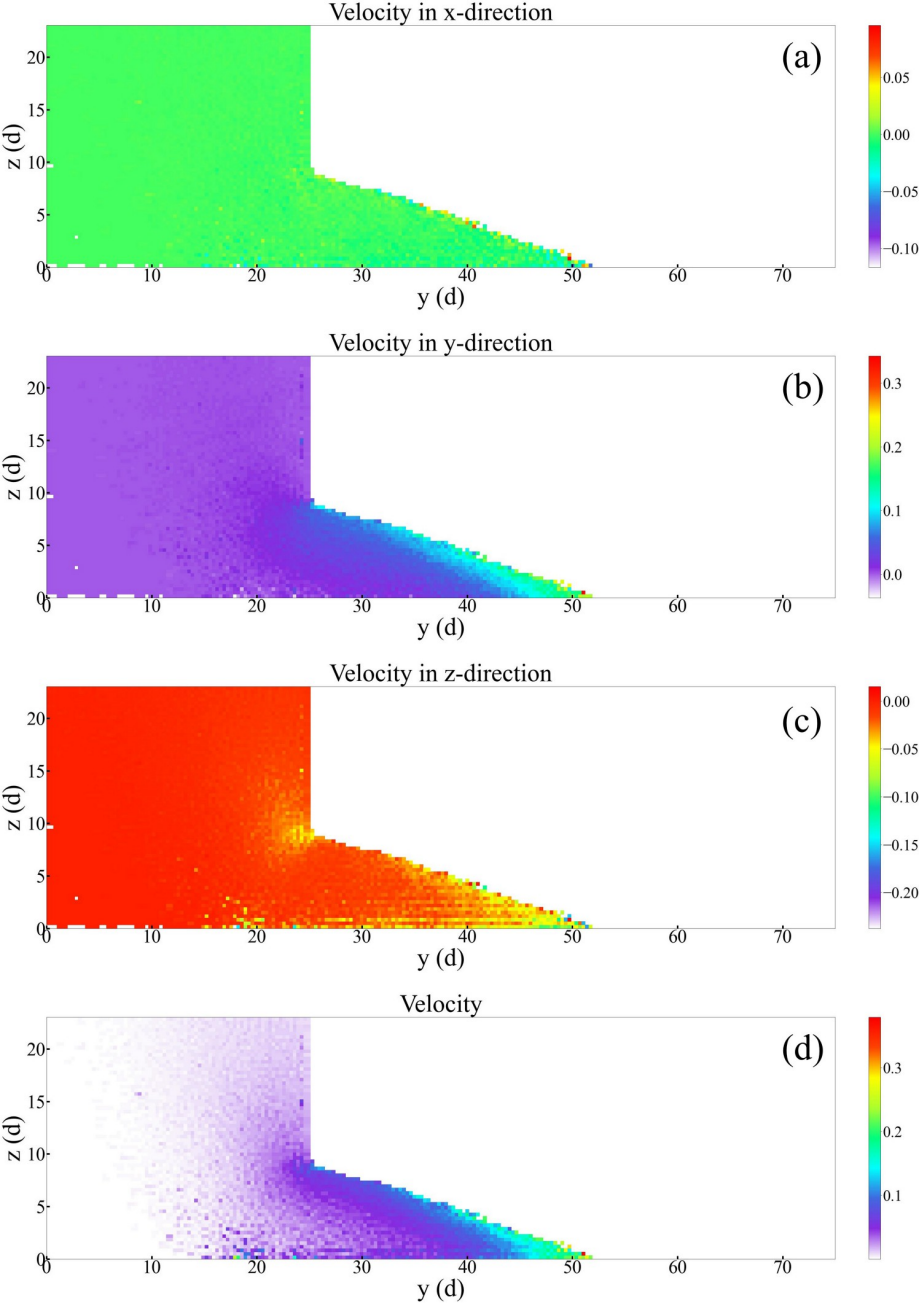
Velocity field diagram of particles inside the slit baffle funnel. The funnel outlet width *W* = 1.5 *d*, the funnel half-angle *θ* = 30°, and the baffle height *H* = 10 *d*. (a) Velocity field in the *x*-direction; (b) velocity field in the *y*-direction; (c) velocity field in the *z*-direction; and (d) total velocity field.

In the previous study, we found that the particle flow rate in the slit baffle funnel varied widely in the *y*-direction. In order to understand the flow pattern of particles inside the slit baffle funnel, we analyzed the velocity field of particles. As shown in Fig 10, the velocity field was plotted under the conditions of funnel outlet width *W* = 1.5 *d*, funnel half-angle *θ* = 30°, and baffle height *H* = 10 *d*. The following four points of information could be obtained from the figure: (1) the velocity of particles in the *x*-direction was close to zero; (2) near the bottom of the baffle, the velocity of particles in the *z*-direction increased; (3) particles flowed out from the bottom of the baffle, forming a surface flow on the right side of the baffle, and the velocity in the *y*-direction gradually increased; (4) the velocity of particles near the left border of the funnel was close to zero, and the particles were clogged.

**Fig 11:**
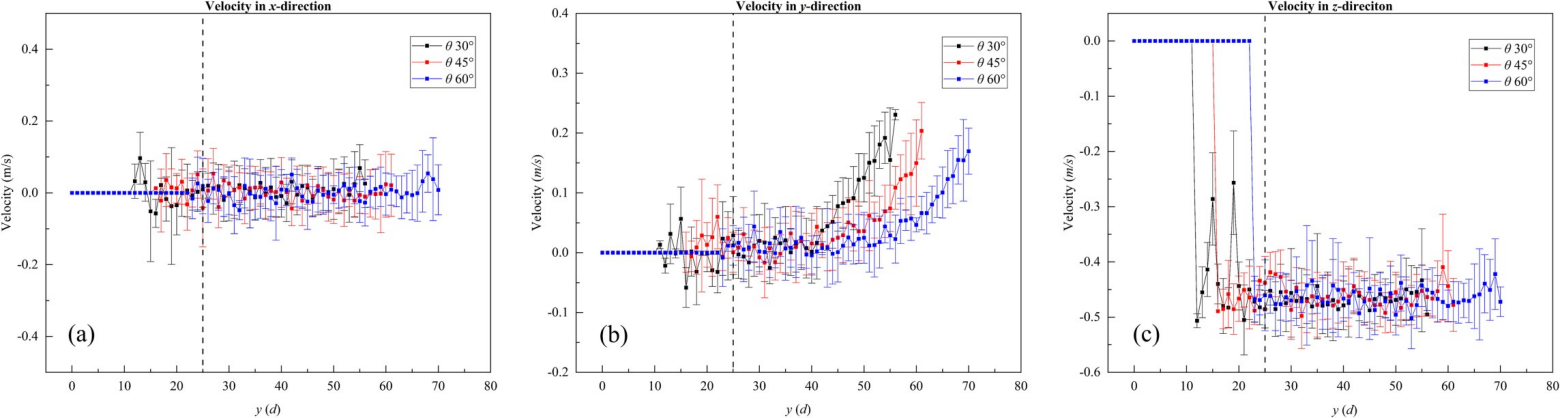
Velocity diagram of particles flowing out of the slit baffle funnel along the *y*-direction. Wherein, the funnel outlet width *W* = 1.5 *d*; the funnel half-angles *θ* are 30°, 45°, and 60° and the baffle height *H* is equal to10 *d*. The black dashed line in the figure indicates the baffle. (a) Outflow velocity in the *x*-direction; (b) outflow velocity in the *y*-direction; (c) outflow velocity in the *z*-direction.

We statistically analyzed the velocity of the outflowing particles along the slit direction (i.e., *y*-direction), at the outlet of the slit baffle funnel, as shown in Fig 11. When *y* was less than 15 *d*, the value of particle velocity was zero and no particles flowed out. As *y* increased, the particle velocity in the *y*-direction gradually increased, but the velocity in the *x*- and *z*-direction was stable and did not change with y.

Figs 10 and 11 shows that when the funnel half-angle *θ* was equal to 30°, the outlet width *W* was less than 1.5 *d*, and the baffle height *H* was equal to 10 *d*, the particles clogged and formed a stagnation zone near the left border region of the funnel, and the flow rate in the funnel was concentrated in the region of *y* > 15 *d*.

### 3.4 Influence of baffle height on flow rate in slit baffle funnels

**Fig 12:**
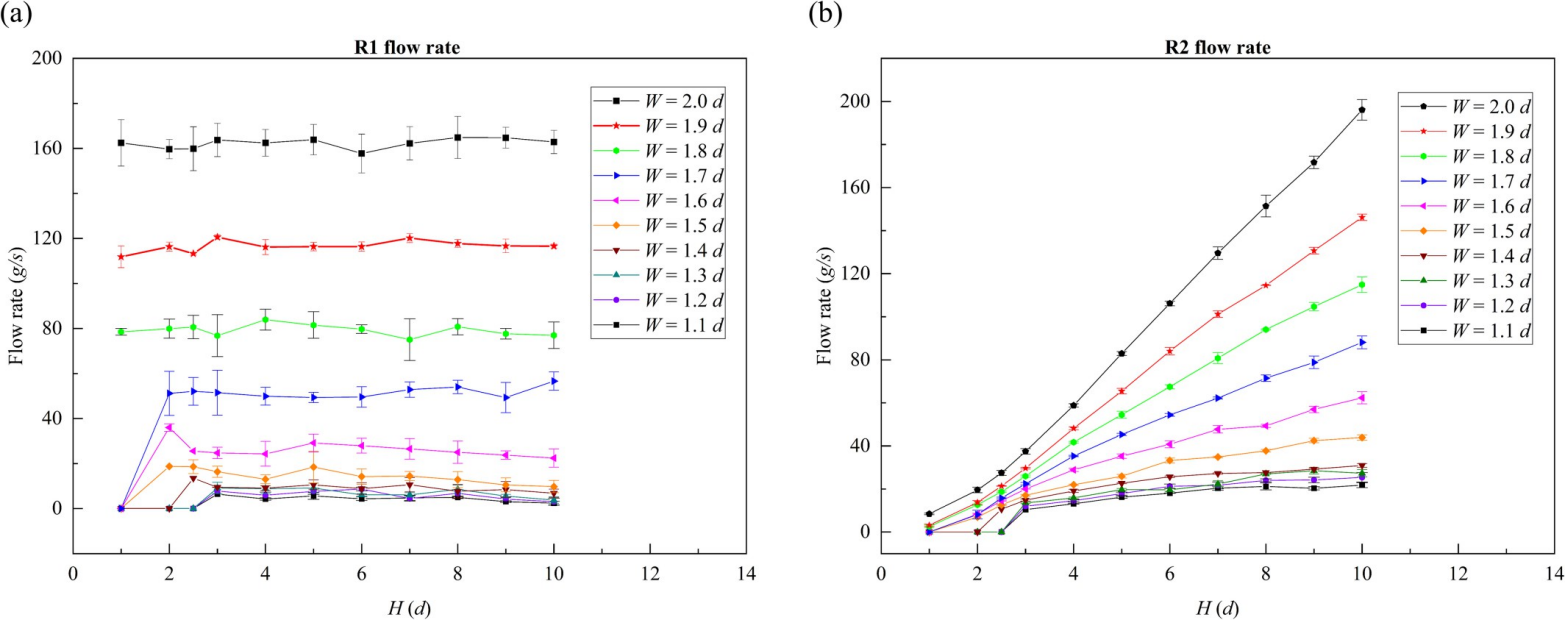
Variation of flow rate with baffle height in a slit baffle funnel. The funnel half-angle *θ* is equal to 30° and the funnel outlet width *W* ranges from 1.1 to 2.0 *d*. (a) Variation of R1 flow rate and (b) variation of R2 flow rate.

We investigated the effect of baffle height *H* on the flow rate, as shown in Fig 12. Fig 12(A) and 12(B) present the variations in the R1 and R2 flow rates, respectively, with the baffle height *H* taking values ranging from 1 to 10 *d*. When outlet width *W* < 1.6 *d* and baffle height *H* < 3 *d*, there were no flow rates in R1 and R2, and the particles in the funnel were clogged. As shown in Fig 12(A), the effect of baffle height *H* on R1 flow rates was not significant when the funnel was not clogged. As shown in Fig 12(B), however, baffle height *H* had a significant influence on R2 flow rate, and the flow rate gradually increased as the baffle height increased. When *H* > 3 *d* and *W* ≤ 1.6 *d*, the R1 flow rate was closed to zero with obvious fluctuations; in contrary, the R2 flow rate was continuous and stable. In the case of *H* = 10 *d*, the R2 flow rate reached 30.95 ± 0.86 *g/s* when *W* = 1.4 *d*, and reached 62.36 ± 2.80 *g/s* when *W* = 1.6 *d*.

**Fig 13:**
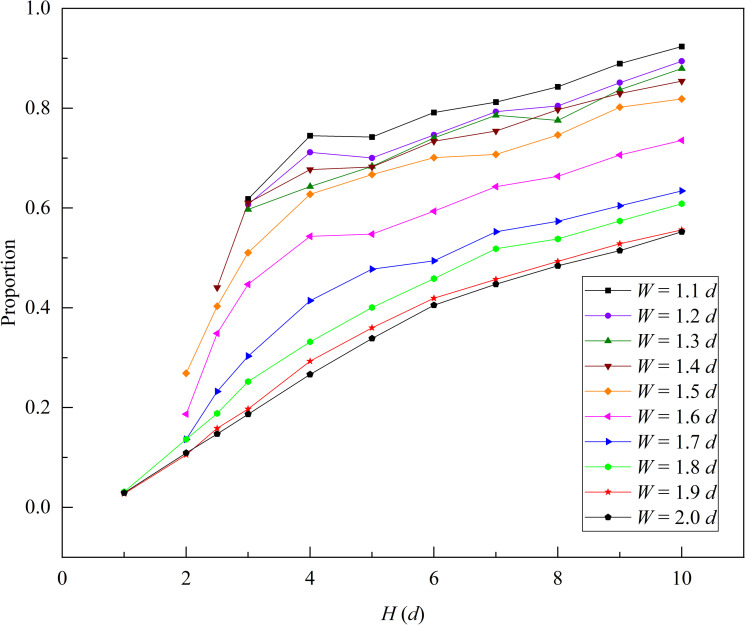
The proportion of R2 flow rate in slit baffle funnel with different baffle heights. Where funnel half-angle *θ* is equal to 30° and the outlet width *W* ranges from 1.1 to 2.0 *d*.

As shown in Fig 13, we analyzed the proportion of the R2 flow rate in the slit baffle funnel at different baffle heights, and found that the proportion gradually increased with the baffle height. When *H* = 10 *d* and *W* ≤ 1.5 *d*, the proportion of R2 flow rate exceeded 0.8.

**Fig 14:**
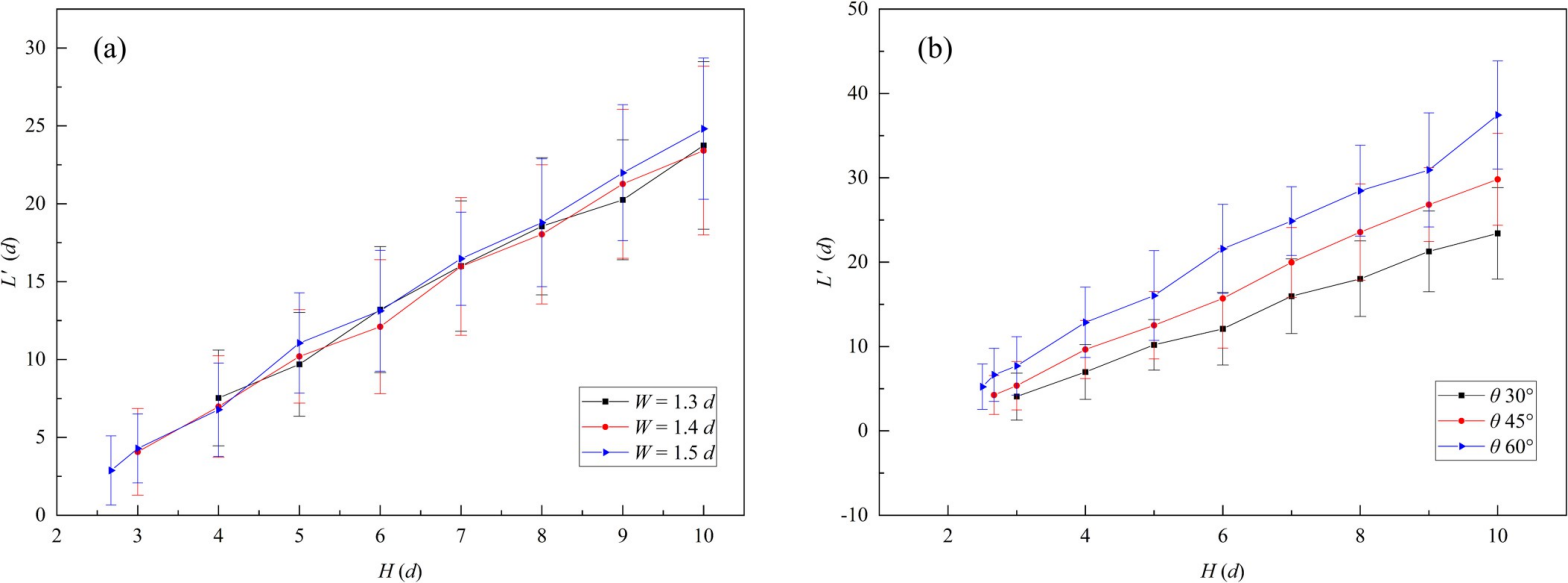
Variation of R2 flow zone length with baffle height in slit baffle funnel. Where *L’* denotes the R2 flow zone length. (a)The funnel half-angle *θ* is equal to 30° and the outlet widths *W* equal to 1.3 *d*, 1.4 *d*, and 1.5 *d*; and (b) the funnel outlet width *W* = 1.4 *d* and the half-angle *θ* are 30°, 45°, and 60°.

The R2 flow zone length *L’* in the slit baffle funnel was investigated at different baffle heights, as shown in Fig 14. As shown in Fig 14(A), the R2 flow zone length *L’* was independent of the outlet width when funnel half-angle *θ* was equal to 30°, and *L’* gradually increased with baffle height. Meanwhile, according to Fig 14(B), the flow zone length *L’* decreased with the funnel half-angle when outlet width *W* = 1.4 *d*.

## 4. Conclusion

In this work, we used DEM method to simulate the dynamic response of mono-size plexiglass particles in two funnel structures. For funnel structure I, slit funnel, when the funnel outlet width *W* > 1.9 *d*, the funnel flow rate conformed to Eq (3), and the flow rate increased as the funnel half-angle decreased. When outlet width *W* < 1.9 *d*, the flow rate was significantly lower than that fitted by Eq (3), and the funnel clogged at *W* < 1.7 *d*. Therefore, we analyzed the clogging probability of the slit funnel and found that the clogging probability *J*(*d*) dropped sharply from 1 to 0 when 1.7 < *W* < 1.9 *d*, and the critical outlet width *W’* of the slit funnel was about 1.9 *d*.

To realize continuous automatic loading of two-dimensional particle experiments and to avoid the clogging problem of slit funnel, we designed the funnel structure II (i.e., the slit baffle funnel) by inserting a baffle inside the slit funnel and divided the funnel into two regions, R1 and R2. We conducted a detail analysis of the flow rate, velocity field, and outflow velocity of the slit baffle funnel with the following conclusions:

1. By inserting the baffle, the particles flowed from the bottom of the baffle to R2, thus solving the clogging problem of the slit funnel. The R1 flow rate was similar to the flow rate in the slit funnel, but R1 clogged when the funnel outlet width *W* < 1.5 *d*. In contrast, the R2 flow rate was stable and reached 24.16 *g/s* at *H* = 10 *d*, *θ* = 30°, and *W* = 1.1 *d*.
2. The proportion of R2 flow rate increased as the funnel outlet width decreased, and when the funnel outlet width *W* < 1.8 *d*, the proportion exceeded 0.6. Meanwhile, the proportion increased with the funnel half-angle.
3. We divided the slit baffle funnel into multiple regions along the *y*-direction with the length unit *l* = 2*d*, and counted the flow rate. We found that the flow rate gradually increased along the *y*-direction and reached the maximum near the flow boundary.
4. We analyzed the particle velocity field in the slit baffle funnel, and found that the velocity in the *x*-direction was close to zero. Particles flowed out from the bottom of the baffle, which increased the velocity in *z*-direction and formed a surface flow, while the velocity in the *y*-direction gradually increased. In R1, where the *y* was small, the particle velocity was close to zero and formed a stagnation zone.
5. We analyzed the velocity at the outlet along the slit direction and found that the velocity in the *x*-direction and *z*-direction was stable, but the velocity in the *y*-direction increased with the slit direction.
6. We analyzed the effect of baffle height *H* on the flow rate in the slit baffle funnel. When *H* > 3 *d*, the funnel had a continuous and stable flow rate, and the baffle height *H* had no effect on the R1 flow rate, but it had a great effect on the R2 flow rate, which increased with baffle height.
7. We analyzed the effect of baffle height *H* on R2 flow zone length *L’*. We found that *L’* increased with the baffle height *H*, but was independent of funnel outlet width. Meanwhile, *L’* was influenced by the funnel half-angle *θ*, and *L’* increased as the funnel half-angle decreased.

In this study, we completed an investigation of flow rate and clogging problem in slit funnels and designed a new funnel structure: a slit baffle funnel. Then, we conducted detail research on the slit baffle funnel in terms of flow rate and velocity field and found that the slit baffle funnel effectively solved the clogging problem of the slit funnel. Most important, the slit baffle funnel had a continuous and stable flow rate with a small outlet width. Therefore, this funnel structure could be used to solve the problem of clogging of particulate matter in the slit geometry in mining, agricultural production, pharmaceutical industry and geology.

## Abbreviations

*d*: Particle diameter, *mm*
*ρ*: Particle density, *g/cm*^*3*^
*υ*: Poisson ratio
*e*: Restitution coefficient
*μ*: Sliding friction coefficient
*θ*: Funnel half-angle, °
*W*: Funnel outlet width, *particle diameter (d)*
*W’*: Critical outlet width, *particle diameter (d)*
*H*: Baffle height, *particle diameter (d)*
*L*_1_: Length of slit funnel, *particle diameter (d)*
*L*_2_: Length of slit baffle funnel, *particle diameter (d)*
*J*(*d*): Clogging probability
*E*: Statistical error
R1: Region1
R2: Region2
*L’*: R2 flow zone length, *particle diameter (d)*
